# Low Prevalence of Liver Disease but Regional Differences in HBV Treatment Characteristics Mark HIV/HBV Co-Infection in a South African HIV Clinical Trial

**DOI:** 10.1371/journal.pone.0074900

**Published:** 2013-12-06

**Authors:** Prudence Ive, William MacLeod, Nompumelelo Mkumla, Catherine Orrell, Ute Jentsch, Carole L. Wallis, Wendy Stevens, Robin Wood, Ian Sanne, Debika Bhattacharya

**Affiliations:** 1 Faculty of Health Sciences, University of the Witwatersrand, Johannesburg, South Africa; 2 Health Economics and Epidemiology Research Office, Department of Medicine, Faculty of Health Sciences, University of the Witwatersrand, Johannesburg, South Africa; 3 Center for Global Health and Development, Boston University, Boston, Massachusetts, United States of America; 4 Department of International Health, Boston University School of Public Health, Boston, Massachusetts, United States of America; 5 Desmond Tutu HIV Centre, University of Cape Town, Cape Town, South Africa; 6 School of Pathology and Wits Health Consortium, Johannesburg, South Africa; 7 David Geffen School of Medicine, University of California Los Angeles, Los Angeles, California, United States of America; Public Health Ontario, Canada

## Abstract

**Background:**

Hepatitis B virus (HBV) infection is endemic in South Africa however, there is limited data on the degree of liver disease and geographic variation in HIV/HBV coinfected individuals. In this study, we analysed data from the CIPRA-SA ‘Safeguard the household study’ in order to assess baseline HBV characteristics in HIV/HBV co-infection participants prior to antiretroviral therapy (ART) initiation.

**Methods:**

812 participants from two South African townships Soweto and Masiphumelele were enrolled in a randomized trial of ART (CIPRA-SA). Participants were tested for hepatitis B surface antigen (HBsAg), hepatitis B e antigen (HBeAg), and HBV DNA. FIB-4 scores were calculated at baseline.

**Results:**

Forty-eight (5.9%) were HBsAg positive, of whom 28 (58.3%) were HBeAg positive. Of those with HBV, 29.8% had an HBV DNA<2000 IU/ml and ALT<40 IU/ml ; 83.0% had a FIB-4 score <1.45, consistent with absent or minimal liver disease. HBV prevalence was 8.5% in Masiphumelele compared to 3.8% in Soweto (relative risk 2.3; 95% CI: 1.3–4.0). More participants in Masiphumelele had HBeAg-negative disease (58% vs. 12%, p = 0.002) and HBV DNA levels ≤2000 IU/ml, (43% vs. 6% p<0.007).

**Conclusion:**

One third of HIV/HBV co-infected subjects had low HBV DNA levels and ALT while the majority had indicators of only mild liver disease. There were substantial regional differences in HBsAg and HbeAg prevalence in HIV/HBV co-infection between two regions in South Africa. This study highlights the absence of severe liver disease and the marked regional differences in HIV/HBV co-infection in South Africa and will inform treatment decisions in these populations.

## Introduction

HIV and hepatitis B virus (HBV) co-infection is common in sub-Saharan Africa with HBV infection in HIV co-infection ranging from 5–17% in South Africa [1], [2]. HIV/HBV co-infection is associated with increased incidence of liver disease and, mortality [3], [4], when compared to HBV monoinfection. Clinical treatment characteristics, which include HBeAg, HBV DNA, ALT, and baseline liver fibrosis, are important predictors of HBV disease progression and are also criteria for HBV treatment initiation. However, these laboratory indicators and how they may vary within populations and are not well characterised in HIV/HBV co-infection in African populations. Neither hepatitis B prevalence nor the distribution of its important clinical characteristics (HBeAg, HBV DNA, or liver fibrosis) may be uniform in sub-Saharan Africa, making application of guidelines that require these measurements a challenge in resource limited settings in Africa. Although HBV is considered endemic (>8%) in this region [5], data in HBV mono-infection demonstrate wide variability's in HBV disease prevalence and its predictors of disease progression in Africa [6].

The markers of particular clinical importance are HBeAg, HBV DNA, ALT, and baseline liver fibrosis. HBeAg is a marker for active HBV replication and elevated HBV DNA levels are associated with cirrhosis and hepatocellular carcinoma [7], [8]. Baseline liver fibrosis is indicative of disease progression. The FIB-4 score, a non-invasive marker for liver fibrosis has shown good sensitivity and specificity [9] for predicting mild and severe liver disease.

As HBV treatment paradigms in HIV co-infection evolve in resource limited settings, it will be important to identify baseline treatment characteristics, including the degree of liver disease, and whether there are regional differences that may influence the timing and initiation of ART in certain populations.

This study sought to identify baseline characteristics and their regional variation, including those characteristics indicative of disease progression and for the initiation of HBV therapy: HBeAg status, HBV viremia, ALT, and liver fibrosis in HIV/HBV co-infected patients initiating HIV therapy. We also sought to compare baseline characteristics in those with and without HBV co-infection. We analysed data from 812 participants from the CIPRA-SA ‘Safeguard the household’ study, a randomised controlled trial of ART monitoring strategies in a resource limited setting, whose primary objective was to evaluate HIV outcomes as a function of HIV care provided by nurses compared to doctors [10].

## Methods

### Ethics Statement

The parent study was approved at the institutional review boards of the University of Witwatersrand and the University of Cape Town. Written informed consent was obtained from all participants before the initiation of study procedures in the parent study [10]. This current post-hoc analysis was performed on stored specimens and this stored specimen and database analysis was approved by the institutional review boards at the University of California, Los Angeles (UCLA) and the ethics committee at the University of the Witwatersrand, South Africa.

### Study population and testing

This prospective study enrolled 812 participants over a two year period starting in February 2005 who were randomised to the nurse or doctor group. Participants were enrolled at two primary health-care sites. The Soweto Township is a more urbanised community with population estimates of 1.3 million. Masiphumelele in Cape Town is a peri-urban township established in 1992, currently home to 17,000 people. Participants were ≥18 years of age, had a CD4+ T-cell count <350 cells/mm^3^ or a previous AIDS defining illness, had no active opportunistic infections at the time of enrolment and were ART naive (excluding previous single dose NVP exposure and/or <28 days of AZT exposure).

On enrolment CD4 count, HIV RNA, biochemistry (including AST, ALT) and haematology studies were performed. In addition, all participants were tested for hepatitis B surface antigen (HBsAg), hepatitis B surface antibody (Anti-HBs), hepatitis B e antigen (HBeAg), hepatitis B core antibody (Anti-HBc) with the Abbott AxSYM Micro-particle Enzyme Immunoassay (MEIA) (Wiesbaden, Germany) platform for Anti-HBs, Anti-HBc, Anti-HBe, HBeAg, HBsAg and HBV DNA, where sample was available. The FIB-4 score was used to assess liver fibrosis. A score of <1.45 has a 90% predictive value of excluding advanced fibrosis [9].

**Figure 1:**
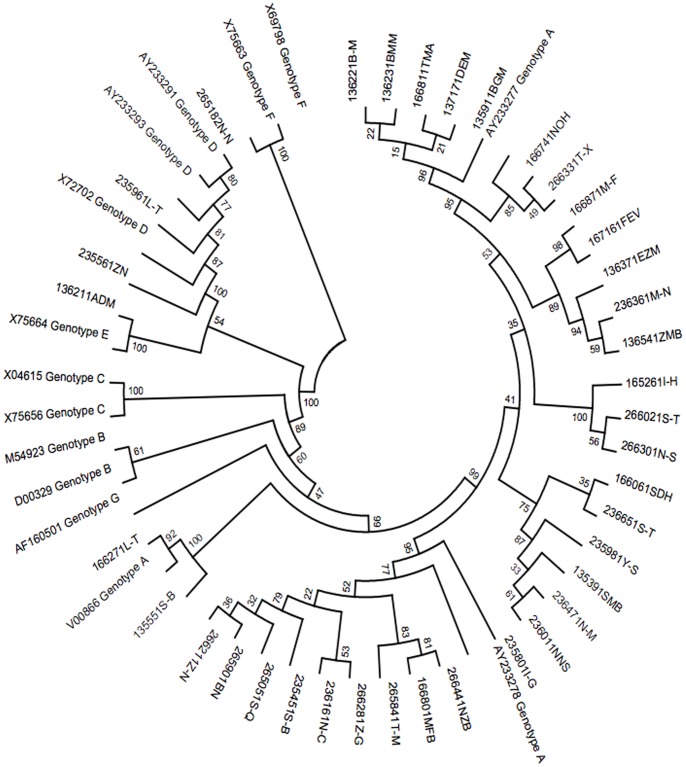
Phylogenetic Analysis of the 37 study entry samples (six digit number, two letters) with HBV pol sequence results and Genbank reference sequences (accession number genotype) at baseline. From this tree 32 sequences cluster with the reference genotype A sequences, four cluster with the genotype D reference sequences and one with the genotype E reference sequences. Bootstrap values on the tree referred to how rooted the phylogenetic tree is at the branch i.e. level of confidence. Note that samples identified as occult HBV infection are excluded from this analysis. 37 isolates were sequenced at baseline, one isolate was sequenced after baseline and was thus not included in the tree.

HBV DNA was tested using the Abbott RealTi*m*e HBV assay (Abbott Park, IL) a polymerase chain reaction (PCR) assay. Results were reported in IU/ml and the lower limit of detection of this assay was 10 IU/ml. Samples with viral loads >60 IU/ml had the *pol* region sequenced. HBV drug resistance testing was performed using a laboratory developed sequencing assay. This assay amplified and sequenced the polymerase gene of HBV [11]. The sequences were analysed using Sequencher version 4.8 and HBV drug resistance and genotype determined using the HBVseq tool on the Stanford HIV drug resistance database (http://hivdb.stanford.edu/HBV/HBVseq/development /HBVseq.html). To verify the genotypes obtained with the HBVseq tool, sequences were further aligned with reference sequences of HBV genotype A-G (GenBank reference sequences; Figure 1), using the Clustal W alignment option within MEGA version 5.10 [12]. On completion of alignment, neighbour-joining phylogenetic tree analysis was performed in MEGA version 5.10 [12], using the p-distance bootstrap model. The stability of the nodes was assessed by bootstrap analysis (100 replicates), and bootstrap values greater than 70% were considered confident [13].

Definitions: The term HBV infection is defined as a positive HBsAg test. The term occult HBV infection is defined as a negative HBsAg test but presence of HBV DNA [14], [15]. These two groups (HBV infection and occult HBV infection) were analyzed separately as those with occult HBV infection may have different clinical outcomes, when compared to HBV infection (defined as a positive HBsAg test).

### Statistical Analysis

We compared continuous variables using T-tests or the Wilcoxon Rank Sum Test and discrete variables using chi-square tests and log-binomial regression [16] . For continuous variables we assessed normality using the Normal Probability Plot and the Shapiro-Wilk test. We used the T-test for normally distributed variables and the Wilcoxon Rank Sum Test for non-normally distributed variables. All analysis was performed using SAS version 9.1.3 Service Pack 4 (SAS Institute Inc., Cary, NC, USA).

## Results

The study enrolled 812 HIV infected participants, 449 (55.3%) from the Soweto site in Johannesburg and 363 (44.7%) from the Masiphumelele site in Cape Town. 804 (99.0%) of the study participants were black African and 573 (70.0%) were women. The median age in of the overall HIV-infected cohort was 32.0 years and the median CD4 count was 165.0 cells per µL (IQR 110.5 to 229.0 cells per µL) at enrolment. Of the 812 HIV-infected participants, 48 (5.9%) were HBsAg positive. Occult HBV infection (HBsAg negative but detectable HBV DNA) was observed in 12 participants (1.5%).

### Baseline Demographic and Serologic Characteristics in those with HBV Infection (HBsAg positive)

The median age of those with HBV infection was 29.5 years. Forty-four percent (21/48) were male. Excluding occult HBV infection, 51.9% of the cohort demonstrated exposure to HBV with at least one or more present of Anti-HBc, Anti-HBs, HBsAg, or HBV DNA. Only 2.7% of the cohort had serology consistent with vaccination (anti-HBs alone). Of the 48 HBsAg positive individuals, 28/48 (58.3%) were HBeAg positive. There were no differences in age or gender distribution in those with and without HBeAg (Table 1). Individuals with HBeAg-positive HBV infection had lower CD4 counts than those with HBeAg-negative HBV infection, 147.0 and 203.0 cells/mm^3^, but this did not reach statistical significance.

**Table 1:** Baseline Demographic and Clinical Characteristics.

	HIV +	HBsAg+	Occult HBV	P value^1^	HBeAg+	HBeAg −	P value
	(n = 752)	(n = 48)	(n = 12)		(n = 28)	(n = 20)	
Median Age	32.0	29.5	32.0	0.0002	30.5	29.0	0.3
Male (%)	28.5%	43.8%	33.3%	0.0264	42.9%(12/28)	45.0%(9/20)	0.8827
Median CD4 (cells/mm^3^), (IQR)	165.0	150.0	112.5	0.8892	147.0	203.0	0.65
	(111.0–229.0)	(78.0–249.0)	(80–179)		(78.0–226.0)	(77.0–269.0)	
Median HIV viral load (log copies/ml), (IQR)	5.1	5.2	5.2	0.5235	5.0	5.3	0.41
	(4.6–5.6)	(4.8–5.5)	(4.9–5.6)		(4.6–5.5)	(4.9–5.6)	
Median AST, (IQR)	28.0	42.0	32.0	0.0002	47.5	36.5	0.0084
	(23.0–38.0)	(29.0–60.5)	(26.5–88.5)		(37.0–80.5)	(24.5–41.5)	
Elevated AST^2^	22.3%	60.4%	41.7%	0.0001	71.4%	45.0%	0.0689
	(168/752)	(29/48)	(5/12)		(20/28)	(9/20)	
Median ALT (IQR)	21.0	33.0	32.0	0.0001	38.5	30.0	0.0251
	(15.0–30.0)	(22.0–55.0)	(20.0–66.0)		(25.0–63.5)	(19.0–38.0)	
Elevated ALT^2^	14.8%	36.2%	40.0%	0.0001	46.4%	21.1%	0.0788
	(111/752)	(17/47)^2^	(4/10)^2^		(13/28)	(4/19)^2^	
Median HBV DNA (IU/ml). (IQR)	–	3.4×10^7^	2.5×10^2^	0.0001	2.9×10^8^	350.7	0.0001
		(443.0–4.1×10^8^)	(62.0–1.4×10^4^)		(1.0×10^8^–6.0×10^8^)	(107.3–2.7×10^4^)	
HBV VL ≤2000	–	29.2%	72.7%	0.0001	3.6%	45.0%	0.0006
		(14/48)	(8/11)^3^		(1/28)	(9/20)	
Elevated ALT^4^ and HBV VL>2000	–	28.3%	11.1%	0.0001	39.3%	11.1%	0.04
		(13/46)^4^	(1/9)^4^		(11/28)	(2/18)^4^	
Median (IQR) FIB-4 Scores^5^	0.8	0.8	1.0	0.34	0.9	0.8	0.03
	(0.6–1.1)	(0.6–1.1)	(0.8–1.2)		(0.7–1.4)	(0.6–0.8)	
FIB-4 Score <1.45	87.6%	83.0%	66.7%	0.58	75.0%	94.7%	0.20
	(602/687)^5^	(39/47)^5^	(8/12)^5^		(21/28)	(18/19)^5^	

1 Comparing HBsAg-positive to HBsAg-negative subjects.

2 Elevated AST and ALT are defined as AST and ALT>40 IU/ml. ALT values are available for 47/48 HBsAg-positive, 19/20 HBeAg-positive, and 10/12 occult HBV subjects.

3 HBV viral loads available for 11/12 occult HBV subjects.

4 ALT and HBV viral loads available for 46/48 HBsAg-positive subjects, 18/20 HBeAg-negative, and 9/12 occult HBV subjects.

5 FIB-4 score: Fibrosis 4 score, available for 687 HIV-positive but HBsAg-negative, 47/48 HBsAg-positive subjects, and 19/20 HBeAg-negative subjects.

### Baseline Virologic and Liver Disease Characteristics in those with HBV Infection (HBsAg positive)

Seventy per cent (33/47) of HIV/HBV co-infected participants had an HBV DNA>2000 IU/ml, only 28.0% (13/46) met criteria for HBV treatment by one standard definition, the combination of any elevation in ALT and HBV DNA≥2000 IU/ml [17]. Of those with HBeAg-negative disease, 45.0% (9/20) had HBV DNA levels below 2000 IU/ml and only 11.1% (2/18) had an elevated ALT and HBV VL≥2000 IU/ml. Overall, 83.0% (39/47) HIV/HBV co-infected individuals had a median FIB-4 score of less than 1.45, indicative of minimal to little liver fibrosis. 94.7% (18/19) HBeAg-negative participants had FIB-4 scores of less than 1.45. Median ALT, AST, and FIB-4 scores were also higher in those with HBeAg positive disease (Table 1).

### Comparison between those with and without HBV Infection (HBsAg postive)

HBV infection was more prevalent in men than in women with 44.0% (21/48) of participants being male in those infected with HBV as compared to 28.5% (214/*752*) in those without HBV (p<0.03). Individuals with HIV/HBV co-infection had lower CD4 counts, 150.0 vs 165.0 cells/mm^3^, but this did not reach statistical significance. HIV-1 viral loads were not different between participants with and without HBV co-infection. Median FIB-4 scores were also not different between HBV infected and uninfected individuals, 0.78 vs. 0.77 (p = 0.34). Median ALT and AST levels were higher in those with HBV infection; 33.0 IU/ml vs. 21.0 IU/ml (p = 0.0001) and 42.0 vs. 28.0 IU/ml, (p = 0.0001) respectively.

### Regional Differences

There were substantial differences in HBV infection: the prevalence in north-central South Africa in Soweto was 3.8% compared to south western South Africa, Masiphumelele, where the prevalence was 8.5% (relative risk 2.3; 95% CI: 1.3–4.0). Despite a higher prevalence of HBV infection, more participants in Masiphumelele had HBeAg-negative chronic HBV (58% vs. 12%; p = 0.002), a type associated with less severe disease. Differences in HBeAg status were also reflected in HBV DNA levels; the median HBV DNA in HBeAg+ and HBeAg (−) HBV was 2.9×10^8^ and 3.5×10^2^ IU/ml, respectively (p<.0001) and median HBV DNA levels between sites was 2.7×10^8^ and 5.8×10^5^ IU/ml in Soweto and Masiphumelele, respectively. Twenty nine and eight tenths percent (29.8%) of all HIV/HBV co-infected participants had HBV DNA levels ≤2000 IU/ml and 33% of participants had HBV DNA levels ≤20,000 IU/ml. However, more HIV/HBV co-infected participants in Masiphumelele had HBV DNA levels ≤2000 IU/ml (43% vs. 6%; p<0.007) and more participants in Soweto had both an elevated ALT and HBV DNA≥2000 IU/ML (35% vs. 24%), but this did not reach statistical significance. There was no difference, however, in median FIB-4 scores between HIV/HBV co-infected participants between treatment sites (Table 2).

**Table 2:** Regional Differences in Clinical Characteristics of HBV-Infected.

	Soweto	Masiphumelele	All Participants	p value
	(n = 17)	(n = 31)	(n = 48)	
Median Age	31.0	29.0	29.5	0.97
Male (%)	35.3%	48.4%	43.8%	0.3838
Median CD4 (cells/mm^3^) (IQR)	195.0	145.0	150.0	0.2405
	(118.0–276.0)	(32.0–246.0)	(77.5–248.5)	
Median HIV viral load (log copies/ml) (IQR)	4.9	5.4	5.2	0.0516
	(4.5–5.2)	(4.8–5.6)	(4.8–5.5)	
HBeAg (+)	88.2%	41.9%	58.3%	0.0051
	(15/17)	(13/31)	(28/48)	
Median AST (IQR)	43.0	41.0	42.0	0.8082
	(35.0–59.0)	(28.0–62.0)	(29.0–60.5)	
Elevated AST^1^	58.8%	61.3%	60.4%	0.8673
	(10/17)	(19/31)	(29/48)	
Median ALT (IQR)	33.0	32.5	33.0	0.3093
	(22.0–66.0)	(21.0–54.0)	(22.0–55.0)	
Elevated ALT^1^	35.3%	36.7%	36.2%	0.9258
Median HBV DNA (IU/ml) (IQR)	2.7×10^8^	5.8×10^5^	3.4×10^7^	0.0067
	(1.2×10^8^–4.4×10^8^)	(2.7×10^2^–2.6×10^8^)	(4.4×10^2^–4.1×10^9^)	
HBV DNA≤2000	5.9%	30.0%	21.3%	0.0169
	(1/17)	(9/30)^2^	(10/47)^2^	
Elevated ALT^1^ and HBV VL>2000	35.3%	24.1%	28.3%	0.4224
	(6/17)	(7/29)^3^	(13/46)^3^	
Median (IQR) FIB-4 Scores^2^	0.8	0.8	0.8	0.93
	(0.6–1.1)	(0.6–1.2)	(0.6–1.1)	
FIB-4 Score <1.45	88.2%	79.3%	82.6%	0.64
	(15/17)	(23/29)^4^	(38/46)^4^	

1 Elevated AST and ALT are defined as AST and ALT>40 IU/ml.

2 HBV viral loads available for 30/31 subjects from Masiphumelele and 47/48 total HBsAg-positive subjects.

3 ALT and HBV viral loads available for 29/31 subjects from Masiphumelele and 46/48 total HBsAg-positive subjects.

4 FIB-4 score: Fibrosis 4 score, available for 29/31 subjects from Masiphumelele and 46/48 total HBsAg-positive subjects.

### Molecular Characteristics

Of the 48 participants that were HBsAg positive, 38 were successfully sequenced during the study, genotype A was the predominant genotype and occurred in 87% (33/38) of cases, followed by genotype D at 10% (4/38), genotype E was observed in one subject. In Soweto all the genotypes identified were genotype A apart from a single case of genotype E. In Masiphumelele the participants were both genotype A and D. There was no HBV drug resistance, as measured by consensus sequencing, at baseline. Figure 1 depicts the phylogenetic relatedness of the HBV isolates.

### Comparison between HBV genotypes and baseline characteristics

Median CD4 was lower in those with HBV genotype A, compared to those with non-A genotypes, (147.0 vs 191.0 cells/mm^3^) but this did not reach statistical significance. Conversely, FIB-4 score was higher in those with non-genotype A (median 2.4 vs 0.8, p = 0.06) (Table 3).

**Table 3:** HBV Genotypes and Baseline Characteristics.

	HBV Genotype A	Non-A HBV Genotype	p value
	(n = 33)^1^	(n = 5)^1^	
Median Age (years)	30.0	32.0	0.3406
	(27.0–32.0)	(29.0–33.0)	
Male (%)	42.4%	40.0%	0.9185
	(14/33)	(2/5)	
Median CD4 (IQR)	147.0	191.0	0.7969
	(75.0–236.0)	(109.0–195.0)	
Median HIV Viral Load (log copies/ml) (IQR)	5.2	5.9	0.3482
	(4.8–5.4)	(4.8–5.9)	
HBeAg positive (%)	69.7%	80.0%	0.6392
	(23/33)	(4/5)	
Median AST (IQR)	43.0	52.0	0.2966
	(30.0–62.0)	(44.0–108.0)	
Elevated AST^2^	57.6%	80%	0.3565
	(19/33)	(4/5)	
Median ALT (IQR)	32.0	65.0	0.5489
	(22.0–54.0)	(21.0–95.0)	
Elevated ALT^2^	33.3%	60.0%	0.2646
	(11/33)	(3/5)	
Median HBV DNA (IU/ml) (IQR)	2.2×10^8^	1.14×10^6^	0.2032
	(6.2×10^6^–4.4×10^8^)	(236.1–2.6×10^8^)	
HBV VL< = 2000	18.2%	20.0%	0.2807
	(6/33)	(1/5)	
Elevated ALT and HBV VL>2000	30.3%	40.0%	0.6654
	(10/33)	(2/5)	
Median FIB-4 Scores^3^ (IQR)	0.8	2.4	0.0596
	(0.6–1.1)	(0.9–2.5)	
FIB-4 Score^3^ <1.45	87.9%	40.0%	0.024
	(29/33)	(2/5)	

1 38 of 48 isolates were successfully sequenced, samples identified as occult HBV infection were excluded from this analysis.

2 Elevated AST and ALT are defined as AST and ALT>40 IU/ml.

3 FIB-4 score: Fibrosis 4 score.

### Occult HBV infection

In this cohort, 12 individuals with occult HBV infection were identified; these individuals had HBV DNA in the absence of any serologic marker for HBV. The median CD4 was 112.5. Median HBV viral load was 2.5×10^2^ IU/ml and median FIB-4 score was 1.0.

## Discussion

In a South African HIV-1 treatment-naïve cohort, HIV/HBV co-infected participants had low levels of liver disease, as measured by FIB-4 scores. Additionally, despite low CD4 counts (CD4<350), only 30% of individuals had both an HBV DNA>2000 IU/ml and elevated ALT which meet the treatment criteria as proposed by Soriano [17]. There were also substantial regional differences in HBV/HIV co-infection and its baseline treatment characteristics in Southern Africa. These factors may impact clinical decision making in HIV/HBV co-infection individuals in sub-Saharan Africa, especially in those populations where assessment of liver disease and HBV viral loads are important to clinical decision-making.

Our study shows differences in baseline predictors of liver disease when compared to Asian, European, or US cohorts, where HBeAg prevalence and HBV DNA levels are higher in HIV/HBV coinfected individuals [18] [19]. Other cohorts with higher enrolment from African sites, however, have shown similar lower prevalences of HBeAg-positive disease and low HBV DNA levels [20]–[22]. Thio and colleagues demonstrated that 66% of patients had HBV DNA<2000 IU/ml [20] and a Nigerian study demonstrated lower HBV DNA levels than US and European cohorts [22]. One explanation may be a difference in HBV genotypes; in one study HBV genotypes D,E, and F were associated with HBeAg-positive disease [20]. Another reason may be the timing of acquisition of infection; in African sites, most infections are thought to arise from horizontal transmission and these infections may be associated with a higher degree of HBeAg clearance, and subsequently, lower HBV viral loads.

The finding of regional differences in sub-Saharan Africa has also been noted in HBV monoinfection and HIV/HBV co-infection but this is the first large study to demonstrate such differences in HIV/HBV co-infection within one country, where HBV treatment guidelines are likely to be standardized. In South Africa, regional differences in have been reported in HBV monoinfected and HIV/HBV co-infected cohorts; low prevalences of HBV infection have been reported in Johannesburg in HIV/HBV co-infection [23] while HBV infection prevalence in an HIV uninfected population in the Eastern Cape, in both urban and rural areas, was 10.4% [24]. Others have found that HBsAg prevalence is higher in rural areas [25], [26]. Such regional variations may be attributable to both age at acquisition and difference in HBV genotypes. Alternatively, practices associated with HBV transmission, such as scarification, may be more common in rural areas.

Current WHO recommendations for the treatment of HIV/HBV co-infection are for initiation of antiretroviral therapy for those with chronic active HBV infection [27]. This definition is evolving but one guideline recommends its definition as an HBV DNA level ≥2000 IU/ml and an elevated ALT [17]. Our data demonstrate that up to 30% of HIV/HBV co-infected individuals in a South African clinical trial have low HBV DNA levels, 88% have low levels of fibrosis, and that there is regional variation in HBV disease. These findings will impact populations where regional variations exist and WHO recommendations for the treatment of chronic active hepatitis are followed, which may require testing for HBeAg and HBV DNA. These findings will also impact cohorts where low HBV viral loads and presence/absence of significant liver disease may change treatment decisions. Such populations include pregnant women, where HBV viral loads are predictors of perinatal HBV transmission and the long term data on the safety of in utero exposure to TDF are still being studied, and HIV/HBV coinfected individuals with preexisting or emerging renal disease who may not have access to other HBV therapies such as entecavir or pegylated interferon. Future questions include the optimal timing of initiation in HIV/HBV co-infected individuals and a longitudinal examination of populations with low fibrosis scores and low HBV DNA levels in African HIV/HBV co-infected populations.
